# The Influence of Lockdown Due to the COVID-19 Pandemic on Weight Management in Patients With Obesity in Portugal

**DOI:** 10.7759/cureus.42036

**Published:** 2023-07-17

**Authors:** Dora Gomes, Raquel Pinto, Elisa Veigas, Catarina R Silva, Miguel L Mendes, Sofia Camões, Catarina Oliveira, Jorge Correia, Maria C Coelho

**Affiliations:** 1 Internal Medicine, Centro Hospitalar Tondela-Viseu, Viseu, PRT; 2 Nephrology, Centro Hospitalar Tondela-Viseu, Viseu, PRT; 3 Internal Medicine, Centro Hospitalar Tondela Viseu, Viseu, PRT

**Keywords:** feeding behavior, mental health, exercise, covid-19, obesity

## Abstract

Introduction: Obesity is a chronic and multifactorial disease, and the COVID-19 pandemic and lockdown have led to changes in the lifestyle habits of those patients. This study aimed to compare compliance with dietary and lifestyle measures, physical activity, mental health status, and motivation to lose weight during the pandemic in these patients.

Methods: An observational study was conducted, which included 63 patients over 18 years old who were followed in the outpatient setting through obesity medical appointments and who agreed to answer a questionnaire.

Results: We found that the majority of patients lost weight during the pandemic (61.7%), associating it with a 68% change in dietary habits. Regarding physical activity, there was a 34.9% reduction in its practice among those who used to exercise before the pandemic. Moreover, 52.4% felt more anxious and 44.4% felt sadder during the lockdown.

Discussion: The weight loss reported in our study seems to be related to a decrease in the consumption of carbohydrates and snacks and a lower percentage of those who relied on takeaways. Additionally, we hypothesize a greater number of home-cooked meals. Regarding exercise, the closure of gyms and the limitations imposed on daily life appear to have contributed unfavorably to this matter. Home confinement, loneliness, and a lack of social activities had harmful effects on the mental health of our sample.

Conclusion: Overall, the hostile influence of the pandemic on mental well-being and exercise habits was evident. Nevertheless, contrary to our expectations, we observed weight loss during the lockdown.

## Introduction

Obesity is a chronic, complex, and multifactorial disease that has become a public health problem worldwide. Its exponential growth stems from a set of worrying lifestyles, including sedentary lifestyles, poor eating habits, irregular working hours, as well as psychosocial risk factors, such as work stress and other emotional stress factors.

It is known that overweight (body mass index, BMI ≥25 kg/m^2^) and obese (BMI ≥30 kg/m^2^) patients have an increased risk of developing cardiovascular disease (namely hypertension, atherosclerosis, and coronary heart disease) and cerebrovascular disease and are at a high risk of mortality compared to the population without the disease [1,2].

The SARS-CoV-2 pandemic surprised the entire world at the end of 2019. In order to reduce the transmissibility of the virus and the number of infections, it was necessary to implement exceptional measures that impacted the habits and lifestyles of the populations [1-3]. The need for social distancing and confinement led to the closure of gyms and other sports complexes, reaffirmed the concept and practice of telework, and led to substantially longer stays at home.

The primary objective of this study was to understand how the SARS-CoV-2 pandemic and the necessary confinement to fight it influenced the weight control of overweight and obese patients. Secondarily, we aimed to analyse the changes in diet and physical activity and determine which psychosocial factors may have contributed more to the changes found.

## Materials and methods

This retrospective and longitudinal study had eligibility criteria based on the follow-up at obesity appointments of patients aged 18 years or older.

All patients enrolled in the study were previously being followed in obesity consultations in a Hospital Center, and recruitment was carried out during these pre-scheduled appointments. Anthropometric data from the period before the lockdown were retrieved from the patient's clinical process.

The study was conducted between July and August 2021.

Questionnaire

We collected data from a questionnaire divided into two groups. The participant has to complete the first 31 questions, mostly dichotomous or multiple-choice. The second part, with eight questions to be filled in by the physician, related to clinical elements such as comorbidities, weight, BMI, and therapy.

We considered two time periods, defined as "before the pandemic" (the date prior to March 18, 2020, coinciding with the first confinement) and "during the pandemic" (from March 18, 2020, to the date of implementation of the questionnaire).

The questions addressed in the first group of the questionnaire included: (i) socio-economic factors, gender, age, employment status, and housing; (ii) smoking habits and alcohol consumption; (iii) number of meals per day prior to and during the confinement; (iv) frequency of recourse to ordering/takeaway from restaurants and fast-food chains; (v) frequency of consumption of selected products (fruit and vegetables, protein sources, carbohydrates, snacks) prior to and during the confinement; (vi) maintenance of eating habits, in case of modification, reasons justifying the change; (vii) physical exercise before and during the confinement; (viii) Evaluation of psycho-emotional state during the confinement.

Ethical aspects

The questionnaire was anonymous and confidential, and participation was voluntary. All participants conveyed their verbal informed consent to participate in the study.

Statistical analysis

Microsoft Excel (version 16.54, Microsoft® Corp., Redmond, WA) for Mac was used for data collection. Data were processed using SPSS.25 software (SPSS Inc., Chicago, IL), where the following significance levels were used: p > 0.05 - not significant; p <0.05 - significant; p <0.01 - highly significant; p <0.001 - highly significant.

In the statistical analysis, we used non-parametric tests, namely Mann-Whitney's U-tests and Kruskal-Wallis tests. The relationships between the variables were tested with a probability of 95%, which results in a significance level of 5% (a=0.05). The decision criteria for testing relationships between variables are based on the study of probabilities, confirming the relationship if the probability is less than 0.05 and rejecting it if it is greater than this value.

## Results

Demographic characterisation

A total of 63 participants were included in the study, of whom 85.7% (n=54) were female and 14.3% (n=9) were male. The participants' ages ranged from 19 to 74 years, with a mean age of 48.9 ± 10.8 years. With regard to cohabitation before and during the pandemic, the majority lived with their partners and children in the city.

Education-wise, the majority of participants (28.6%) had completed secondary education. Full-time employment was the dominant occupational status. In terms of employment during the pandemic, the majority (71.4%) reported maintaining their usual job (Table 1). However, it should be noted that there was an increase in unemployment from 27% to 28.6%, as well as an increase in sick leave.

**Table 1:** Sociodemographic characterization of the sample according to gender

Variable	Male		Female		Total	
	N (9)	% (14.3)	N (54)	% (85.7)	N (63)	% (100.0)
Education						
Without	0	---	1	1.9	1	1.6
Year 1–4	1	11.1	11	20.4	12	19.0
Year 5–6	2	22.2	4	7.4	6	9.5
Year 7–9	2	22.2	16	29.6	18	28.6
High school	1	11.1	13	24.1	14	22.2
Bachelor	2	22.2	6	11.1	8	12.7
Master	1	11.1	2	3.7	3	4.8
PhD	0	---	1	1.9	1	1.6
Employment situation before the pandemic						
Unemployed	1	11.1	16	29.6	17	27.0
Part-time	0	---	1	1.9	1	1.6
Full-time	7	77.8	29	53.7	36	57.1
Student	0	---	2	3.7	2	3.2
Sick leave	0	---	0	---	0	---
Retired	1	11.1	6	11.1	7	11.1
Affected the job						
Stay the same	6	66.7	39	72.2	45	71.4
Mandatory telework	---	---	3	5.6	3	4.8
Telework and face-to-face work	2	22.2	0	---	2	3.4
Became unemployed	---	---	3	5.6	3	4.8
Started during the pandemic	1	11.1	6	11.1	7	11.1
Sick leave/retired	---	---	3	5.6	3	4.8

Characterisation of toxicophilic habits

Regarding smoking habits, seven of the respondents smoked before the pandemic, and one person quit smoking during the pandemic. Of the respondents who smoked, we found that, on average, during the pandemic, there was an increase in the number of cigarettes smoked per day, from 10 to 12.

Regarding alcohol habits, 81% of the participants stated that they had not consumed alcohol previously and continued to abstain from consuming it. Of those who did drink alcohol, wine consumption increased from an average of 1.56 glasses/day to 1.63 glasses/day during the pandemic. Beer consumption remained unchanged, with an average of one beer per day.

Characterisation of eating habits

The average number of daily meals increased from 4.65 meals per day to 5.17 meals per day during the pandemic, with the maximum number of meals reported being eight before the pandemic and nine during the pandemic. Regarding takeaway food, 76.2% of respondents stated that they had never ordered it (Table 2). Notably, a higher percentage of men (33.3%) reported ordering takeaways more frequently compared to women (1.9%). During the pandemic, men reported ordering food 1.89 times per week, while women reported ordering food 0.35 times per week.

**Table 2:** Characterization of takeaways, changes in eating habits, and consumption of some food groups, depending on gender ^a^Multiple answer question

Variable	Male		Female		Total	
	N (9)	% (14.3)	N (54)	% (85.7)	N (63)	% (100.0)
Takeaway						
The same	1	11.1	7	13.0	8	12.7
Ordered more	3	33.3	1	1.9	4	6.3
Ordered less	1	11.1	2	3.7	3	4.8
Never ordered	4	44.4	44	81.5	48	76.2
Change of habits						
Maintain habits	5	55.6	18	33.3	23	36.5
Changed habits	4	44.4	36	66.7	40	63.5
Changes in eating habits^a^						
Larger portions	3	33.3	8	14.8	11	17.4
Less healthy eating	1	11.1	9	16.7	10	15.9
More healthy eating	4	44.4	23	42.6	27	42.9
Pre-cooked meals	2	22.2	3	5.6	5	7.9
Reason for changing eating habits^a^						
More time to cook	1	11.1	9	16.7	10	15.9
Less time to cook	1	11.1	5	9.3	6	9.5
More time for socializing	0	---	5	9.3	5	7.9
Anxiety and stress	2	22.2	13	24.1	15	23.8
Calm and relaxation	0	---	3	5.6	3	4.8
More access to food	2	22.2	9	16.7	11	17.5
Less portion control	2	22.2	4	7.4	6	9.5
I ate out more	1	11.1	10	18.5	11	17.5
Night craving	0	0	3	5.6	3	4.8
Motivated to eat healthily	2	22.2	11	20.4	13	20.6
Consumption of certain food groups						
Fruits and vegetables						
Ate the same	5	55.6	31	57.4	36	57.1
Eat more	3	33.3	16	29.6	19	30.2
Eat less	1	11.1	7	13.0	8	12.7
Protein source						
Ate the same	6	66.7	37	68.5	43	68.3
Eat more	1	11.1	5	9.3	6	9.5
Eat less	2	22.3	12	22.2	14	22.2
Carbohydrates						
Ate the same	4	44.4	22	40.7	26	41.3
Eat more	1	11.1	7	13.0	8	12.7
Eat less	4	44.4	24	44.4	28	44.4
Don't usually eat	0	---	1	1.9	1	1.6
Snacks						
Ate the same	2	22.2	13	24.1	15	23.8
Eat more	2	22.2	7	13.0	9	14.3
Eat less	0	---	21	38.9	21	33.3
Do not usually eat	5	55.6	13	24.1	18	28.6

Most participants reported changes in their eating habits, with 66.7% of women and 44.4% of men answering affirmatively. The consumption of fruits, vegetables, and protein sources remained the same. However, 44.4% of participants reported a decrease in carbohydrate intake. Regarding snacks, women had a higher consumption habit than men, although 38.9% of women reported a decrease in snack consumption during the pandemic (Table 2).

With regard to the reasons given for the abovementioned changes, we highlight, in the case of men, negative influencing factors, such as anxiety and stress (22.2%), access to food (22.2%), and less control over the portions eaten (22.2%), while on the other hand, 22.2% reported greater motivation for healthier eating. In the case of women, anxiety and stress were also the most frequently mentioned modifying factors (24.1%), followed by a greater motivation for healthier eating (20.4%) and eating out more often (18.5%).

Characterisation of physical activity habits

In terms of physical exercise, the majority of participants (74.6%) reported regular practice (Table 3). No statistically significant differences were found (p=0.813) between genders in terms of exercise practice. When asked about changes in physical activity during the confinement period, 34.9% of all respondents reported a decrease in physical activity. However, 44.4% of men maintained their usual practice.

**Table 3:** Characterization of physical exercise before the pandemic and changes in physical exercise during it, according to gender ^a^Multiple answer question

Variable	Male		Female		Total	
	N (9)	% (14.3)	N (54)	% (85.7)	N (63)	% (100.0)
Physical exercise						
No	2	22.2	14	25.9	16	25.4
Yes	7	77.8	40	74.1	47	74.6
Changes in physical exercise						
Maintain	4	44.4	15	27.8	19	30.2
Practiced less	3	33.3	19	35.2	22	34.9
Practiced more	0	---	6	11.1	6	9.5
Continued not to practice	1	11.1	8	14.8	9	14.3
Started to practice	1	11.1	6	11.1	7	11.1
Decision to change physical exercise habits^a^						
Worry less about weight loss	1	11.1	3	5.6	4	6.3
Less motivation	2	22.2	8	14.8	10	15.9
More motivation	1	11.1	12	22.2	13	20.6
Gyms closed	1	11.1	6	11.1	7	11.1
More time	0	---	7	7.13	7	11.1
Less time	1	11.1	3	5.6	4	6.3
Had no company	0	---	5	9.3	5	7.9
Other	0	---	5	9.3	5	7.9

Weightlifting was the modality on which respondents reported spending the most time, and this time increased during the pandemic. The practice of running and cycling also increased, while time spent walking and in group classes decreased. With regard to the reasons for changing exercise habits during the pandemic (Table 3), 20.6% reported feeling more motivated, although 15.9% reported the exact opposite (less motivated). Less preoccupation and less free time were also highlighted (11.1%). Most respondents reported that the most common place for physical exercise was outdoors, both before and during the pandemic (63.5% and 66.7%, respectively). There was a decrease in attendance at gyms, from 11.1% to 1.6%, and an increase in physical exercise at home, from 9.5% to 15.9%. Of the respondents, 61.9% did not seek help for weight loss during the pandemic. It is noteworthy that, of those who sought help, 10 went to a physician, nutritionist, or pharmacist; 6 went to a personal trainer; and 1 did it through the Internet.

Characterisation of mood

In terms of the emotions expressed during the confinement period, anxiety (52.4%), sadness (44.4%), and a lack of patience (44.4%) were the most commonly mentioned feelings. Feelings of accomplishment and happiness were less frequent, at 7.9% and 6.3%, respectively (Table 4). Among those who mentioned sadness, only four patients sought professional help (one from a physician and three from a psychiatrist).

**Table 4:** Characterization of feelings expressed during confinement ^a^Multiple answer question

Feelings^a^	N (63)	% (100.0)
More sad	28	44.4
More anxious	33	52.4
Happier	4	6.3
Want to cry	12	19.0
Most accomplished	5	7.9
Less patience	28	44.4
Depressed	15	23.8

Characterisation of anthropometric data

In general terms, we found an average weight reduction of 5.73 kg for those involved from the phase before the pandemic to confinement, with a BMI reduction of 2.30 kg/m^2^ (Table 5). We also found that the weight reduction is more noticeable in women (6.14 kg) than in men (3.44 kg).

**Table 5:** Statistics on weight and BMI by gender, before and during the pandemic

		Male			Female			Total		
		Before	After	Difference	Before	After	Difference	Before	After	Difference
Mean	Weight (kg)	111.56	108.11	3.44	103.00	97.49	6.14	104.28	99.03	−5.73
	BMI (kg/m^2^)	35.78	34.89	0.89	39.14	36.91	2.55	38.63	36.61	−2.30
Deviation	Weight (kg)	16.65	18.93	14.63	17.73	19.57	14.12	17.70	19.69	14.11
	BMI (kg/m^2^)	3.87	4.86	3.95	6.65	7.77	5.70	6.40	7.42	5.48
Minimum	Weight (kg)	90	88	−15	76	60	−15	76	60	−15
	BMI (kg/m^2^)	29	28	−5	28	23	−5	28	23	−5
Maximum	Weight (kg)	142	147	37	158	173	58	158	173	58
	BMI (kg/m^2^)	43	44	9	55	60	21	55	60	21

Of the patients surveyed, 23.8% were proposed for metabolic surgery, and 31.7% had already undergone the surgery. Most (66.7%) of the participants were medicated with drugs used to aid weight loss. In this study, we found that patients used drugs considered off-label for the treatment of obesity, such as fluoxetine, metformin, sodium-glucose cotransporters inhibitors (SGLT2) inhibitors, and GLP-1 agonists. However, 30.2% of the patients had type 2 diabetes mellitus and 28.6% had depressive syndrome, pathologies that by themselves could justify the use of these drugs (Table 6).

**Table 6:** Characterization of proposed patients or who underwent metabolic surgery and therapy carried out with the aim of losing weight, depending on gender

Variable	Male		Female		Total	
	N (9)	% (14.3)	N (54)	% (85.7)	N (63)	% (100.0)
Proposed for surgery						
No	8	88.9	40	74.1	48	76.2
Yes	1	11.1	14	25.9	15	23.8
Performed surgery						
No	7	77.8	36	66.7	43	68.2
Yes	2	22.2	18	33.3	20	31.7
Medication for obesity						
No	4	44.4	17	31.5	21	33.3
Yes	5	55.6	37	68.5	42	66.7

After conducting the descriptive analysis of the obtained data, as written above, an inferential approach was taken using analytical statistics. For the respondents who changed their habits, there was a greater percentage of weight loss compared to those who did not change their habits, with statistically significant differences (p=0.019).

As regards food consumption of some food groups before and during the confinement, namely carbohydrates, we highlighted a domain of weight loss in those who reported having eaten less, followed by those who kept the same consumption, with quite significant statistical differences (p=0.006). Also in relation to the variation in the consumption of snacks during the pandemic, there was a domain of weight loss in those who reported having eaten less, followed by those who did not usually eat it, with statistically significant differences (p=0.030).

Regarding physical exercise during the pandemic, we found that the participants who kept practicing were the ones who showed greater weight loss, followed by those who started to practice, and finally, those who started to practice more. Those who practiced less and those who continued not to practice were the ones who revealed less weight loss. However, this difference is not statistically significant (p=0.392).

Regarding the influence of bariatric surgery on weight variation, those who underwent surgery showed greater weight loss than those who did not, with this variation presenting very significant statistical differences (p=0.001). With regard to the pharmacological treatment of obesity, we found that in the respondents without medication, there was greater weight loss, however, without significant statistical differences (p=0.572).

## Discussion

During the SARS-CoV-2 pandemic, measures of confinement and social isolation were imposed in all societies around the world, with Portugal being no exception [1-6]. The closure of all non-essential establishments, where gyms were included, and the limitation on physical exercise even in the open-air spaces of large cities had major consequences for the mental and physical health of the entire population. Moreover, the need to mobilise human resources in hospitals for the targeted and effective combating of COVID-19 led to the cancellation of most non-urgent medical appointments.

Our results showed that, contrary to expectations, most study participants lost weight during the pandemic (61.7%), with a more marked mean reduction in females than in males (6.14 kg and 3.44 kg, respectively), although not a statistically significant result. This weight loss seems to be related to the change in eating habits since 68% of the population reported changes, considering that they follow a healthier diet (p<0.05). There was a lower consumption of carbohydrates and snacks [6] among those who already had the habit of eating them (p<0.01 and p<0.05, respectively), and 28.6% of the respondents did not have the habit of eating snacks. These data are similar to the results obtained in a study carried out in the Spanish population, in which an improvement in diet during confinement was registered by the preference for a more Mediterranean diet, with lower consumption of red meat, sweets, and fizzy drinks and higher consumption of fruit and vegetables [7], the same as identified in some participants of an Italian study [6]. In addition, it was clear that most of our patients did not order takeaway food during confinement (76.2%). With the closure of shopping centres and restaurant spaces, there was also an important decrease in meals in these establishments, so it is hypothesised that there was greater cooking of homemade meals, and this may also have contributed to a better diet (hypothesis also put forward in the study published by Ferrante et al. [4]).

Regarding the practise of physical exercise, most of our sample had already developed the habit of practising sports, 34.9% of which reduced their frequency. On the other hand, a greater amount of time spent on weight-lifting exercises was registered during the pandemic [6], as well as an increase in running and cycling. The most reported reasons for changes in this practise are somewhat contradictory, with 20.6% of respondents saying they felt more motivated and 15.9% less motivated. We may relate these changes to the closure of gyms [1] and the limitations applied to the mobility of the population even in outdoor spaces where they would normally practise sports [4]; on the other hand, the fact of having more time and more motivation to maintain the practise of physical exercise and the impossibility of going to gyms may have positively influenced the habit of this practise at home. However, these data were not statistically significant in relation to weight loss.

Regarding mental health, the unfavourable influence of the pandemic and the constraints it implied were evident among the participants. More than half of the sample reported feeling more anxious (52.4%) and 28 participants (44.4%) more sad, and of these, only 4 sought professional help. These data are not surprising and are corroborated by other published studies [8-10]. The confinement at home, the loneliness, and the lack of social and leisure activities contributed to the increase of these feelings, as well as the financial uncertainty resulting from the instability of the professional situation [11].

Another factor to be taken into account is the performance of bariatric surgery in our sample. Among the respondents who underwent gastric sleeve or gastric bypass, there was a greater weight loss compared to those who did not undergo surgery, with a statistically significant difference (p=0.001), which is in line with what would be expected regarding the surgical treatment of obesity.

This study once again highlighted the impact of the pandemic and the measures adopted on the habits and lifestyles of the population, with a special focus on obese patients. Although the results obtained were not in line with other studies published to date, we emphasise that our questionnaire was applied only to patients with documented obesity and with follow-up in specialised appointments, unlike many of the published studies, in which the analysis is carried out in the general population [12,13], with and without the metabolic disease.

Finally, regarding possible limitations of our study, we highlight the fact that the questionnaire applied is completed by each participant and is subject to interpretation and subjectivity by each one, which may create a bias in the results obtained.

## Conclusions

This study clearly demonstrates the unfavourable impact of the SARS-CoV-2 pandemic on the mental health and physical exercise habits of obese patients. While we observed a significant percentage of patients who achieved weight reduction during this period, it is important to note that there were also patients who experienced weight gain, putting them at an increased risk of mortality, as is widely known. Obesity has been recognised as one of the major pandemics of the 21st century, with significant morbidity and mortality rates globally. Therefore, it is crucial to emphasise the importance of continuous monitoring for these patients, even during outbreaks and epidemics. Furthermore, we need to reconsider how we can effectively provide ongoing care in an era increasingly prone to new pandemics.
